# Optimizing antimicrobial routes: risk factor analysis for intravenous therapy in children with preseptal orbital cellulitis

**DOI:** 10.1186/s13052-025-01931-x

**Published:** 2025-06-15

**Authors:** Roberto Privato, Emanuela Inserra, Francesco Pezzoli, Alessia Nucci, Stefano Masi, Elena Chiappini, Giuseppe Indolfi, Sandra Trapani, Luisa Galli, Elisabetta Venturini

**Affiliations:** 1 Department of Health Sciences, University of Florence, Florence, Italy; 2 Pediatric Emergency Department, Meyer Children’s Hospital IRCCS, Florence, Italy; 3 Paediatric Infectious Diseases Unit, Meyer Children’s Hospital IRCCS, Florence, Italy; 4 Pediatric Unit, Meyer Children’s Hospital IRCCS, Florence, Italy; 5 Department of NEUROFARBA, University of Florence, Florence, Italy

**Keywords:** Antimicrobial route, ASSET score, Cellulitis, Children

## Abstract

**Background:**

The optimal route of antimicrobial administration for preseptal orbital cellulitis (OC) in children remains uncertain. While mild cases may be managed with oral therapy, distinguishing between mild and severe presentations is challenging. The recently proposed ASSET score offers a tool for assessing the severity of skin and soft tissue infections, but prospective validation in large cohorts of preseptal OC is lacking. As a result, most patients with periorbital infections are admitted for intravenous (IV) antibiotics. This study aims to identify clinical and laboratory features predictive of severe preseptal OC requiring IV treatment.

**Methods:**

A retrospective study was conducted at Meyer Children’s Hospital IRCCS, Florence, reviewing outpatient records for OC cases from January 2017 to June 2024. Data on age, sex, clinical presentation, blood tests, and management were collected.

**Results:**

Previously initiated oral therapy was associated with the need for IV therapy (*p* < 0.001), as well as the presence of fever (*p* < 0.001), and severe eyelid swelling (*p* < 0.001). The median ASSET score was higher in patients with preseptal OC requiring IV therapy (*p* < 0.001). Differences in laboratory findings were noted between children with preseptal OC managed with and without IV antibiotics.

**Conclusions:**

Previous trials of oral antibiotics, systemic features, and severe swelling influence clinicians’ decisions to initiate IV antibiotics in preseptal OC. If validated for preseptal OC, the application of the ASSET score could significantly reduce the number of children treated with IV antibiotics. Ancillary blood tests may be useful for detecting preseptal OC requiring IV treatment.

## Introduction

The incidence of orbital cellulitis (OC) in children is estimated at 1.6 per 100,000 cases per year [1]. It can be classified as either preseptal or postseptal, depending on whether the infection is confined to the anterior portion of the orbit or extends beyond the orbital septum, a thick connective tissue structure separating the superficial orbital region from the rest of the orbital cavity [2]. OC may develop from the spread of infection from nearby structures or through exogenous and endogenous sources [3]. Disruptions in the skin barrier, such as trauma or insect bites, are more commonly associated with preseptal OC [4]. Postseptal OC is strongly linked to paranasal sinusitis and is clinically characterized by proptosis, diplopia, or decreased visual acuity [5]. However, periorbital swelling could often make difficult to distinguish between these two conditions. In children, most cases are limited to the preseptal space and have a favorable prognosis [6]. Nevertheless, untreated preseptal infections can lead to intracranial complications if not addressed promptly. While empirical intravenous (IV) antimicrobial therapy is the treatment of choice for postseptal cellulitis, there is currently insufficient evidence regarding the optimal route of administration for preseptal cellulitis [7]. Moreover, authors have reported that empirical oral antimicrobial treatment is as safe and effective as IV therapy [8]. The American Academy of Pediatrics recommends that mild cases of preseptal cellulitis can be managed with oral antibiotic therapy and daily follow-up until significant improvement is observed. Mild preseptal cellulitis has been defined as cases in which the eyelid is less than 50% closed [7]. However, this definition has limitations, as eyelid closure cannot be measured with accuracy and may, therefore, vary according to the clinician’s judgment. In addition, imaging studies and laboratory tests rarely play a role in the management of preseptal cellulitis [9]. In fact, despite they are frequently performed in clinical practice, studies demonstrating their ability to distinguish between mild and severe forms or proving their role in guiding the therapeutic choice, are lacking. Therefore, distinguishing between mild and severe preseptal cellulitis in young children could be challenging. As a result, most patients with periorbital infections are admitted for IV antibiotics [10]. However, inpatients management of children with mild periorbital cellulitis is an unfavorable cost-effective strategy, considering also the risks associated with hospital length of stay [11]. Recently, Ibrahim et al. introduced the ASSET score as a tool to assess the severity of skin and soft tissue infections and to identify patients requiring IV antibiotics [12]. The ASSET score consists of five items (eye involvement, systemic features, swelling, tenderness, and body surface area affected). It is suggested that patients with an ASSET score of less than three, particularly those without systemic features and who have not had a trial of oral antibiotics, could be managed with oral antibiotics [13]. However, prospective validation in large cohorts of preseptal cellulitis is still needed, and evidence defining mild disease remains limited. This retrospective study aims to identify clinical and laboratory features predictive of severe preseptal cellulitis requiring IV treatment.

## Methods

### Data collection

A retrospective study was carried out at a tertiary-level pediatric hospital (Meyer Children’s Hospital IRCCS) in Florence. The pediatric emergency department (PED) database was screened for all children presenting with OC from January 1st, 2017, to June 1st, 2024. Two independent investigators reviewed outpatient clinic records for the ICD-9 code related to OC (376.01). Any discrepancies were resolved by consensus or by consulting a senior infectious disease specialist. Inclusion criteria were as follows: age < 18 years, diagnosis of preseptal OC, availability of complete clinical documentation. Preseptal OC was defined as clinically documented OC without radiological or clinical evidence of postseptal involvement, including proptosis, restricted ocular motility, and vision changes. Data collected included: age, sex, clinical presentation, therapy administered prior to PED admission, blood test results at admission, route and duration of antibiotic therapy, hospital length of stay. ASSET score was retrospectively derived from the initial evaluation performed upon admission to the PED, as documented in the medical records. The severity of the swelling was retrospectively abstracted verbatim based on the treating physician’s qualitative assessment (mild or severe). The data were entered into the study database in accordance with international standards for the protection of information and respect for privacy.

### Statistical analysis

Quantitative variables consistent with a normal distribution were represented as the mean ± standard deviation (SD). Data not conforming to a normal distribution were represented by the median and interquartile ranges (IQRs). Categorical variables were described as numbers (n) and percentages (%). The Mann-Whitney U non parametrical test was used to compare continuous patient characteristics. Statistical differences between categorical variables were analyzed using the χ2 test. Multivariate logistic regression was performed for factors identified as significant in the univariate analysis. The Hosmer and Lemeshow goodness-of-fit test was applied. Unadjusted and adjusted odds ratios with their 95% confidence intervals (CIs) were calculated. Data was managed and analyzed using IBM SPSS Statistic (version 28th). *P* < 0.05 was considered as the statistically significant threshold.

## Results

The selected ICD-9 code identified a total of 238 cases of OC during the study period, of those 93% (223/238) were preseptal OC. Demographic and clinical characteristics of the study population are summarized in Table 1, considering the route of administration of the antibiotic treatment.

Overall, 17% (37/223) of the enrolled children had already started oral antibiotic therapy prior to their clinical evaluation in the emergency department, with a median duration of 2 days (IQR 1–4); in all these cases, amoxicillin-clavulanate had been administered. The use of non-steroidal anti-inflammatory drugs (NSAIDs) was reported in 6% (14/223) of cases. The median hospital length of stay of children with preseptal OC was 5 days (IQR 2–7), and the median duration of IV antibiotic therapy was 5 days (IQR 2–8).

A longer interval between symptom onset and evaluation at emergency department was found in children with preseptal OC who subsequently received IV therapy (median 2 days, IQR 1–3 vs. 1 day, IQR 0.5-2, *p* < 0.001). Moreover, considering only those already on oral antimicrobial therapy, a longer symptoms duration was found in patients admitted for IV therapy (median 3 days IQR 2–4 vs. 1 day IQR 0.5-3, *p* < 0.001). Differences in laboratory findings between children with preseptal OC requiring IV antibiotic therapy and those with preseptal OC not requiring IV therapy are shown in Table 1. In particular, children requiring IV antibiotics had a significantly higher level of absolute white cell count and neutrophils (absolute value and percentage). The median ASSET score was significantly higher in patients with preseptal OC requiring IV therapy (2 IQR 2–3 vs. 3 IQR 3–4, *p* < 0.001). One hundred and eighty out of 222 patients (81%) with preseptal OC had a score < 4. Of these, 46/222 (21%) received IV antibiotics.

**Table 1:**
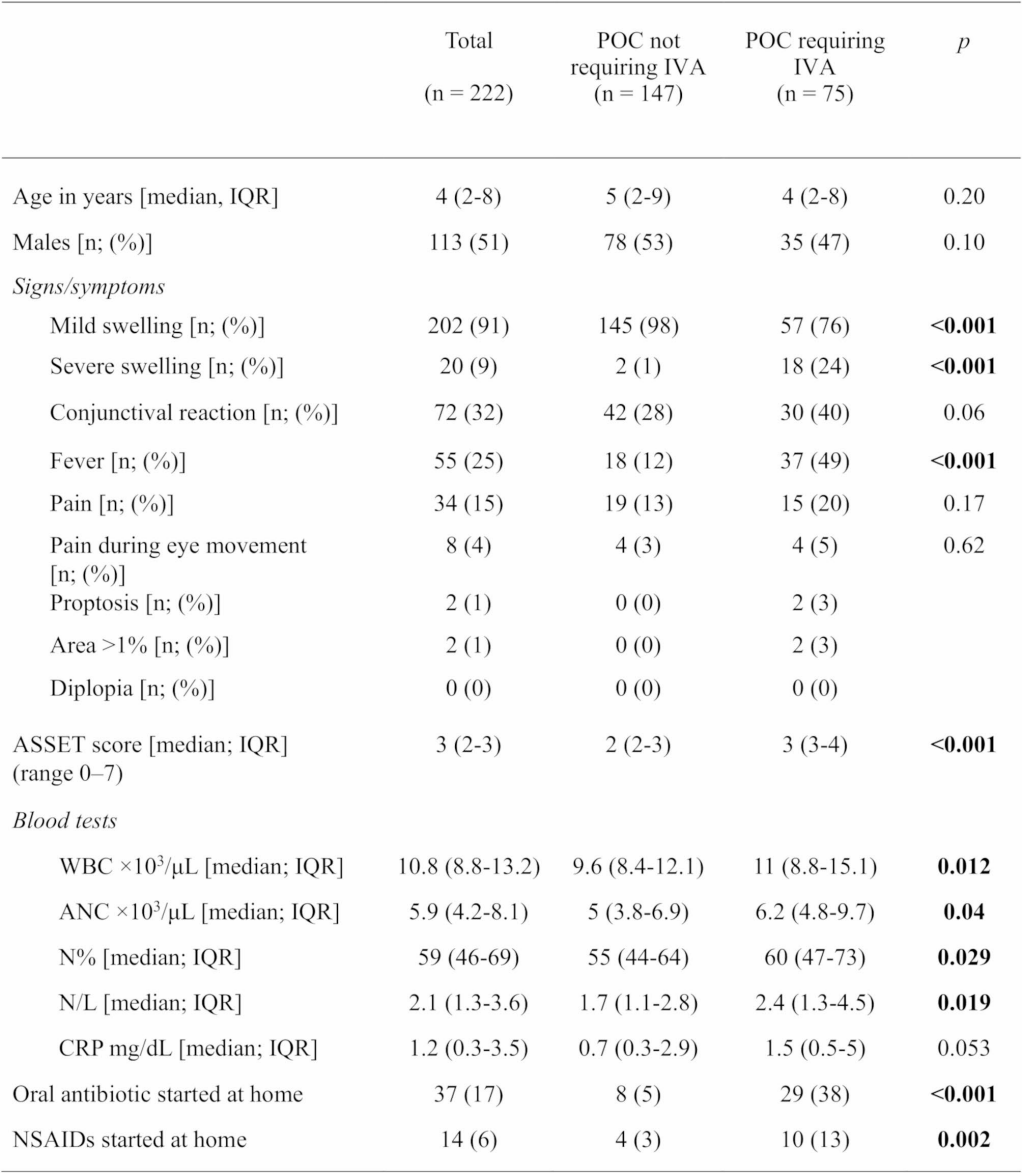
Demographic and clinical characteristics of children presenting to the pediatric emergency department with preseptal cellulitis

PED, pediatric emergency department; POC, pre-septal orbital cellulitis; IVA, intravenous antibiotic therapy; NSAIDs, non-steroidal anti-inflammatory drugs; WBC, total white blood cell count; ANC, absolute neutrophil count; *N*%, percentage of neutrophil; N/L, neutrophil to lymphocyte ratio; CRP, C-reactive protein; *p*, *p* value

In the univariate analysis, the presence of previously initiated oral antibiotic therapy was associated with the need for hospitalization and IV therapy among children with preseptal OC (*p* < 0.001, unadjusted odds ratio [OR] 10.78, 95% CI 4.6–25.5), as well as the presence of fever (*p* < 0.001, OR 6.9, 95% CI 3.5–13.5) and severe eyelid swelling (*p* < 0.001, OR 45.7, 95% CI 5.9–351). These variables remained significant in the multivariate logistic regression analysis (Table 2). The Hosmer and Lemeshow goodness-of-fit test indicated that the data fit the model well (χ² = 5.79, degrees of freedom = 5, *p* = 0.85). Previous administration of NSAIDs was significantly associated with severe swelling (*p* < 0.001). It also correlated with the need for IV therapy in the univariate analysis but did not remain significant at multivariate analysis (Table 2).

**Table 2:** Univariate and multivariate predictors of intravenous antibiotic therapy in children with preseptal cellulitis

	Univariate analysis			Multivariate analysis		
	uOR	CI 95%	*p*	aOR	CI 95%	*p*
Age, years	0.9	0.8–1.04	0.35			
Pain	1.7	0.8–3.5	0.17			
Fever	6.9	3.5–13.5	**< 0.001**	5.5	2.5–12.1	**< 0.001**
Severe eyelid swelling	45.7	5.9–351	**< 0.001**	38	4.7–290	**0.001**
Oral antibiotic started at home	10.8	4.6–25.5	**< 0.001**	9.3	3.6–23.8	**< 0.001**
NSAIDs started at home	5.4	1.6–18	**0.005**	1.1	0.2–5.5	0.87
ASSET score	7.7	3.5–16.7	**< 0.001**	1.8	1.08–3.1	**0.027**

NSAIDs, non-steroidal anti-inflammatory drugs; uOR, unadjusted odds ratio; aOR, adjusted odds ratio; C, confidence interval; *p*, *p*-value

## Discussion

To the best of our knowledge, only few studies have investigated clinical and laboratory predictors for the need for IV therapy in a large cohort of children with preseptal OC. Due to a lack of high-quality evidence, empirical treatment guidelines regarding the optimal antimicrobial route in preseptal OC are largely based on expert opinion. Our study showed that patients who had a trial of oral antibiotics prior to their admission to the pediatric emergency department were more frequently treated with IV antibiotics. This aligns with previous evidence indicating that IV therapy should be initiated whenever oral therapy fails [14]. However, there is currently no validated definition of treatment failure [15]. The Food and Drug Administration recommends assessing clinical response to treatment 48 to 72 h after initiating therapy [16]. Most clinicians consider 48 h as the optimal time frame for determining if treatment failure has occurred following antibiotic initiation [17]. In our sample, the median time frame from symptom onset was longer for patients who were subsequently administered intravenous therapy (*p* < 0.001). Among patients who were already on oral antimicrobial therapy, those who were hospitalized had symptoms for a longer duration (*p* < 0.001). These findings suggest that children with symptoms lasting more than 48 h are at higher risk for treatment failure and disease progression. However, a standardized definition of treatment failure is needed to better guide clinical decision-making.

The presence of a severe infection, based on the clinician’s assessment, plays a key role in deciding the route of antibiotic administration in real-life scenarios [15]. Nevertheless, this approach can be subjective and may vary according to the clinician’s experience. There is limited evidence indicating objective and reproducible parameters of disease severity in preseptal OC.

The Melbourne ASSET score is a risk assessment tool developed to guide clinicians on which patients require IV antibiotics [12, 13]. The ASSET score consists of five items (eye involvement, systemic features, swelling, tenderness, and body surface area affected). Patients with an ASSET score of less than three, particularly if without systemic features and if they didn’t receive a trial of oral antibiotics, could be managed with oral antibiotics [13]. Consistently, our data showed that the presence of fever correlates with the decision to administer parenteral antimicrobial therapy. In fact, the onset of fever in the context of skin and soft tissue infections correlates with disease severity [14]. Moreover, previous studies showed that fever can be a sign of orbital involvement [18]. Therefore, it seems reasonable to consider fever, malaise, and other signs of systemic illness as factors influencing the route of therapy. The presence of severe swelling was also associated with the need for IV therapy, as suggested by previous literature [13]. However, the evaluation of clinical features like swelling may be operator-dependent, and further studies are needed to assess inter-observer reliability. In this paper, the median ASSET score was significantly higher in patients with preseptal OC requiring IV therapy (*p* < 0.001). This aligns with the study by Ibrahim et al., who reported mean ASSET scores in those receiving intravenous treatment compared to those treated with oral antibiotics of 3.9 ± 1.2 vs. 3.0 ± 0.8, respectively [13]. Notably, in our study 180 out of 222 patients with preseptal OC had an ASSET score below 4. Of these, 46 (21%) received IV antibiotics. Therefore, implementation of the ASSET score for preseptal OC in clinical practice could potentially decrease the number of children requiring IV antibiotic treatment. Outpatient treatment strategies may enhance the standard of care in cases of preseptal OC, by reducing costs and minimizing the risks associated with prolonged hospital stays. Additionally, these strategies could also contribute to improved antimicrobial stewardship. In our sample, NSAID administration was associated with more severe eyelid swelling. Due to the retrospective nature of this study, it was not possible to determine whether NSAIDs contributed to the worsening of the condition or if they were administered due to the severity of the initial presentation. However, growing evidence suggests that these drugs may play a role in the development of severe complications from common bacterial infections [19, 20]. Moreover, NSAIDs are known to mask infection signs/symptoms, potentially delaying diagnosis and treatment, which can lead to more severe presentation. In our population, NSAID use was associated with the need for IV therapy in the univariate analysis but lost significance in the multivariate analysis, indicating that other factors played a more prominent role in determining the clinical outcome (Table 2). Larger, prospective randomized studies are needed to establish whether NSAID consumption is a trigger for severe complications and worse outcomes in children with periorbital infections.

As previously mentioned, diagnosis of OC primarily relies on the patient’s history and physical examination. However, in real-world practice, clinical evaluations are often supported by laboratory investigations, especially in patients with severe disease features. In our cohort, laboratory values showed differences between patients treated with oral antibiotics and those treated with IV antibiotics. In particular, the mean leukocyte count was higher in the group requiring IV therapy, as were absolute neutrophil count and percentage of neutrophils. The neutrophil to lymphocyte ratio (NLR) is a relatively novel biomarker of systemic inflammation that relies on complete blood count values. It has shown a promising role in several areas of clinical practice, including sepsis [21]. Our analysis showed significantly higher NLR in patients requiring IV therapy. However, it remains challenging to determine whether these laboratory values genuinely reflect clinical severity or independently influence the decision to initiate IV therapy. To avoid multicollinearity, blood tests were excluded from the logistic regression model, as they could be strongly correlated with the presence of fever and systemic illness. This approach prioritized the influence of clinical parameters on decision-making. Indeed, we postulated that therapeutic decisions were primarily driven by clinical indicators due to the lack of strong evidence supporting the role of laboratory values in guiding treatment routes. Future randomized studies are needed to assess the direct impact of laboratory values on therapeutic decisions.

Our study has limitations. First, as with all retrospective studies, there may have been inaccuracies in data collection and reporting, as well as missing data. Second, this was a single-center study, which could introduce referral bias. Third, due to the retrospective nature of the study, it is possible that some patients discharged from our emergency department with oral therapy may have later been admitted to other care centers or emergency departments due to treatment failure. Nevertheless, this study was conducted at the only tertiary level pediatric hospital in Tuscany, which serves as the referral center for pediatric infectious diseases. None of the children who received oral treatment was readmitted to our hospital with symptoms of OC.

Despite these limitations, our study has several strengths. The observation period was long (8 years), and the analysis was performed on a database from a tertiary pediatric hospital, capturing a large number of patients.

## Conclusions

This study highlights that previous trials of oral antibiotics, the presence of systemic features, and severe periorbital swelling are associated with clinicians’ decisions to initiate IV antibiotics in children with preseptal OC. Ancillary blood tests could be helpful in identifying patients who genuinely require IV treatment but further, prospective studies are needed to confirm their utility. If validated for preseptal OC, the ASSET score could substantially decrease the number of children receiving unnecessary IV antibiotics. These findings emphasize the importance of standardized decision-making tools in minimizing avoidable hospital admissions and optimizing patient management. Future research should focus on expanding the validation of the ASSET score in larger populations and further exploring its integration into clinical practice.

## Data Availability

The datasets used and analysed during the current study are available from the corresponding author on reasonable request.

## Abbreviations

CI: Confidence interval
IQR: Interquartile range
IV: Intravenous
NLR: Neutrophil to lymphocyte ratio
NSAIDs: Non-steroidal anti-inflammatory drugs
OC: Orbital cellulitis
OR: Odds ratio
PED: Pediatric emergency department
